# Mobility and Invasion Related Gene Expression Patterns in Equine Sarcoid

**DOI:** 10.3390/ani10050880

**Published:** 2020-05-19

**Authors:** Przemysław Podstawski, Wojciech Witarski, Tomasz Szmatoła, Monika Bugno-Poniewierska, Katarzyna Ropka-Molik

**Affiliations:** 1 Department of Animal Molecular Biology, Laboratory of Genomics, National Research Institute of Animal Production, Krakowska 1, 32-083 Balice, Poland; wojciech.witarski@izoo.krakow.pl (W.W.); tomasz.szmatola@izoo.krakow.pl (T.S.); katarzyna.ropka@izoo.krakow.pl (K.R.-M.); 2 Department of Animal Reproduction, Anatomy and Genomics, University of Agriculture in Kraków, Mickiewicza24/28, 30-059 Kraków, Poland; monika.bugno-poniewierska@urk.edu.pl; 3 University Centre of Veterinary Medicine, University of Agriculture in Kraków, Mickiewicza 24/28, 30-059 Kraków, Poland

**Keywords:** skin neoplasia, horse, cell physiology, real-time PCR

## Abstract

**Simple Summary:**

The current studies profiled the expression of five equine sarcoid cell genes related to cell mobility and invasion (cell cycle control binding protein alpha, coronin 1b, metalloproteinase 2, tissue inhibitor of metalloproteinases 3 and vimentin) and compared the expression of these genes in healthy skin fibroblasts. Cells were collected from healthy and sarcoid-affected skin biopsies obtained by a qualified veterinarian. Gene expression patterns were investigated under two different conditions of cell culture, with high and low availability of nutritional components in the culture medium. The results showed significant differences in the expression of the two analyzed genes (coronin 1b and vimentin) depending on culture conditions. The obtained results emphasize the complexity of the genomic background of sarcoids and indicate the importance of further research on genes related to the physiological changes that occur in sarcoids.

**Abstract:**

Sarcoids are the most common skin neoplasm in the Equidae family. Sarcoids are benign, but may cause severe damage in affected animals. Due to the high risk of post-treatment recurrence and the lack of an effective method of treatment, it is reasonable to perform studies on the molecular aspects of this neoplasm. Therefore, the present studies analyzed five genes (cell cycle control binding protein alpha, coronin 1b, metalloproteinase 2, tissue inhibitor of metalloproteinases 3 and vimentin) related to cell mobility and invasion traits. Primary healthy fibroblasts and sarcoid cells were obtained from skin biopsies. Cell lines were cultured in two different medium types with different concentrations of foetal bovine serum (10% and 0.5% FBS) to study its influence on the analyzed genes. Gene expression was measured using the real-time PCR method. The results showed significant differences in two genes (coronin and vimentin) depending on culture conditions. In conclusion, the results enabled finding two new genes, related to cell motility and invasion traits, in which gene expression is deregulated. Results of the study may put new knowledge into the complexity of the genetic background of this disease and show the importance of further analysis on this subject.

## 1. Introduction

A sarcoid is generally defined as a non-metastasizing but locally invasive neoplasm that appears in equines regardless of age, coloration or part of the animal body. It is one of the most common skin tumors observed in Equidae. Its occurrence is not officially established, but comprises up to 12% of all Equidae skin conditions, and it can reach a frequency of 90% of all skin tumors [1,2,3]. A sarcoid is a benign skin tumor, and it was considered a cosmetic change for a long time [1]. Despite its tissue-specific character, a sarcoid may affect the animal by decreasing the wellbeing and value of the animal. Notably, the general condition of affected animals improves after recovery from the sarcoid [1]. These studies indicate that this neoplasm affects the animals despite its limited migration ability, likely via the secretion of metabolites [1]. Some reports confirmed that sarcoids can transform into more severe forms, in several cases with mechanical injuries to the affected region. Veterinary trials indicate that the neoplasm goes into recurrence with an estimated probability of 30%, which makes our understanding of sarcoid physiology and the development of new treatment methods an important task [1,4,5].

The exact mechanism that leads to the development of sarcoid neoplasm is not well known. However, *bovine papillomavirus* types 1 and 2 (*BPV* 1 and 2) may cause sarcoid because its DNA is present in most tumor tissue samples [6,7]. The DNA of *BPV* 1 and *BPV* 2 viruses accumulate in epithelial cells [8,9,10]. Although the genomic structure and carcinogenic character of some genes of *BPV* were established [11,12], the molecular influence of the virus on the host genome and its phenotype effect are not well recognized. The genetic mechanism related to sarcoid origination was investigated using gene expression modification [13], miRNA profiling [14], copy number variation and copy neutral loss of heterozygosity occurrence [15]. The research strongly indicates a deregulation of molecular pathways in sarcoids compared to healthy tissue, which may be involved in neoplastic transformation. Semik et al. [13] indicated that 901 pathways may be essential for sarcoid occurrence and growth. Unger et al. [16] showed that proteoglycans in cancer, viral carcinogenesis and Hippo signalling pathways were strongly related to sarcoid formation. The use of novel molecular genetic methods was also used to establish non-invasive diagnostic biomarkers, which may be used for confirmation of sarcoid-affected horses [17]. These authors demonstrated the whole blood and serum miRNA profiles and specific miRNAs that only occurred in affected animals.

Due to the diversity of sarcoid tumors, there are no effective and universal methods of sarcoid treatment. Many treatments are efficient for only one specific type of tumor or tumors in specific locations. For example, intratumoral injections of *Bacillus Calmette* and Guerin (BCG) vaccine into the periorbital area exhibit high effectiveness (from 83% to 100%), but the efficacy is lower (approximately 50%) in other areas. Many treatment methods also produce unwanted effects, such as the recurrence of a more aggressive form in cases of surgical methods, or death of the animal (BCG vaccine) [18].

Notably, sarcoids show no traits of metastasis but invade surrounding skin cells. Some *BPV* genes interfere with the cytoskeleton proteins of host cells [11], which may be a cause of sarcoid cell migration into the surrounding layers of the affected tissue. The metastasis process, which is one of the eight original known hallmarks of cancer, is the major reason for cancer-related death. Metastasis causes up to 90% deaths related to cancer [19,20]. A sarcoid is a tumor that generally has a low metastasis ability, but its probability of recurrence after treatment is high. Analyses of genes related to cell motility and invasion may result in a better understanding of the limited metastasis mechanism of sarcoids. The present study investigated five genes, *CDC42bpα* (cell division control 42 binding protein alpha), *CORO1b* (coronin), *MMP2* (matrix metalloproteinase 2), *TIMP3* (tissue inhibitor of metalloproteinases 3) and *VIM* (vimentin), as potentially associated with cell cytoskeleton remodeling, proliferation, collagen catabolism and cell adhesion.

## 2. Materials and Methods

The experiment was performed on two different cellular mixes of cell lines. To minimalize the effect of the individual cell line, both cell line mixes were prepared out of four (in case of fibroblast cells) or three (in case of sarcoid cells) different cell lines added in an equal cell ratio. Each cell line was obtained from one tissue. Sarcoid primary cell lines were obtained from equine tumor tissues diagnosed by veterinarian by clinical appearance and removed in surgery with a few centimetres of skin margin. There was one sample of nodular sarcoid tissue and two samples of mixed sarcoid tissue used. The primary sarcoid cell line was broblasts weobtained from those samples, after removal of normal skin tissue fragments. Primary cell lines of fire obtained from tissues collected from horse normal skin biopsies collected from croup. There were four skin samples used.

For the fibroblast cells, the protocol was approved by the Animal Care and Use Committee of the Institute of Pharmacology, Polish Academy of Sciences in Cracow (no. 1173/2015). The sarcoid cells were provided during veterinarian procedures of sarcoid removal. The Polish Act on the Protection of Animals Used for Scientific or Educational Purposes of 15 January 2015 (which implements Directive 2010/63/EU of the European Parliament on the protection of animals used for scientific purposes) states that ethic approval by the Animal Ethics Committee is not mandatory for research conducted on biological material provided during veterinarian services.

*BPV* 1 and *BPV* 2 DNA viability was checked in normal skin fibroblast cell lines. Lines in which viral DNA was detected were removed from the experiment due to the probability of a negative impact on the results. *BPV* DNA in normal skin cells could indicate that used tissue was affected by sarcoid which was not recognized by veterinarian. All lines were cultured under standard conditions (37 °C, 100% humidity, 5% CO_2_) in Dulbecco’s MEM High-Glucose (Gibco, Thermo Scientific, Waltham, MA, USA) with 10% foetal bovine serum (FBS, Gibco, Thermo Scientific, Waltham, MA, USA), L-glutamine (GlutaMAX-I, Corning, Wiesbaden, Germany, 10 mL/L), sodium pyruvate (Gibco, Thermo Scientific, Waltham, MA, USA, 110 mg/L) and the antibiotic Primocin (Invitrogen, Thermo Scientific, Waltham, MA, USA, 0.1mg/mL). During the experiment, the cell-mixed lines were divided into two experimental groups. The first group was cultured under the previously mentioned conditions, and the other group was cultured in medium containing 0.5% FBS to introduce signal depravation and inhibit cell proliferation.

Primary cell lines were obtained according to modified procedures of Tomasek et al. [21]. Healthy skin and sarcoid tissue samples were sterilized with 70% ethanol after hair removal. The tissue was cut into small pieces and placed into culture dishes containing an appropriate amount of culture medium. After one week, tissue pieces were removed, and spontaneously migrated cells were cultured into stable lines.

Further analyses required healthy skin samples that were free of *BPV* 1 or 2 DNA. Therefore, viral DNA was isolated using reagents from the NucleoMag Vet kit (Macherey-Nagel, Warszawa, Poland) from fibroblast cells according to the manufacturer’s protocol. The quality of isolated nucleic acids was measured in a NanoDrop 2000 (Life Technology, Thermo Scientific, Waltham, MA, USA). Amplification of isolated DNA (150 ng per probe) was performed using DNA AmpliTaq 360 DNA Polymerase (Life Technology, Thermo Scientific, Waltham, MA, USA) using the reaction conditions described in the manufacturer’s manual. The primers used for viral *E5* gene DNA sequence detection (F: CAAAGGCAAGACTTTCTGAAACAT and R: AGACCTGTACAGGAGCACTCAA) were described by Teifke [22]. PCR products were visualized on a 2.5% agarose gel with the addition of ethidium bromide. Samples that contained *BPV* DNA were removed from subsequent procedures.

Total cell RNA was isolated using the PureLink RNA isolation kit (Ambion, Life Technologies, Thermo Scientific, Waltham, MA, USA), according to manufacturer’s protocol. To avoid contamination of RNA by DNA, the PureLink DNase Set was used (Invitrogen, Thermo Fisher Scientific, Waltham, MA, USA). The quality and quantity of isolated total RNA were measured using 2% agarose gel electrophoresis and spectroscopy with a NanoDrop 2000 (Life Technology, Thermo Scientific, Waltham, MA, USA). cDNA was obtained from 500 ng of total RNA in a reverse transcription reaction using the High Capacity cDNA reverse kit protocol (Applied Biosystems, Thermo Scientific, Waltham, MA, USA).

The recent study investigated five genes: *CDC42bpα* (ENSECAG00000017881.1), *CORO1b* (ENSECAG00000014882.1), *MMP2* (ENSECAG00000000953.1), *TIMP3* (ENSECAG00000018314.1), *VIM* (ENSECAG00000004216.1) and *GAPDH* as an endogenous control. Primer sequences were designed using Primer3 [23]. The primers used are shown in Table 1.

The reaction for each gene was performed in three replications using AmpliQ 5× HOT EvaGreen qPCR Mix Plus (ROX) (BIOTUM, Novazym, Poznań, Poland) according to the manufacturer’s protocol. Analysis was performed in Quant Studio7Flex (Applied Biosystem, Thermo Scientific, Waltham, MA, USA). Obtained data were analyzed using Quant Studio™ 7 Flex Real-Time PCR System Software (Applied Biosystem, Thermo Scientific, Waltham, MA, USA). Transcript levels of the investigated genes were calculated using the ΔΔCT method according to the Pfaffl method [24]. Gene expression was presented in comparison to the sample with the lowest expression level. The real-time PCR method efficiency was evaluated using the standard curve method. Statistical analyses were performed using R software [25]. The normality of the distribution was tested using the Shapiro–Wilk test, and differences between groups were analyzed using the Kruskal–Wallis test.

## 3. Results

### 3.1. Cell Division Control 42 Binding Protein Alpha

**Figure 1:**
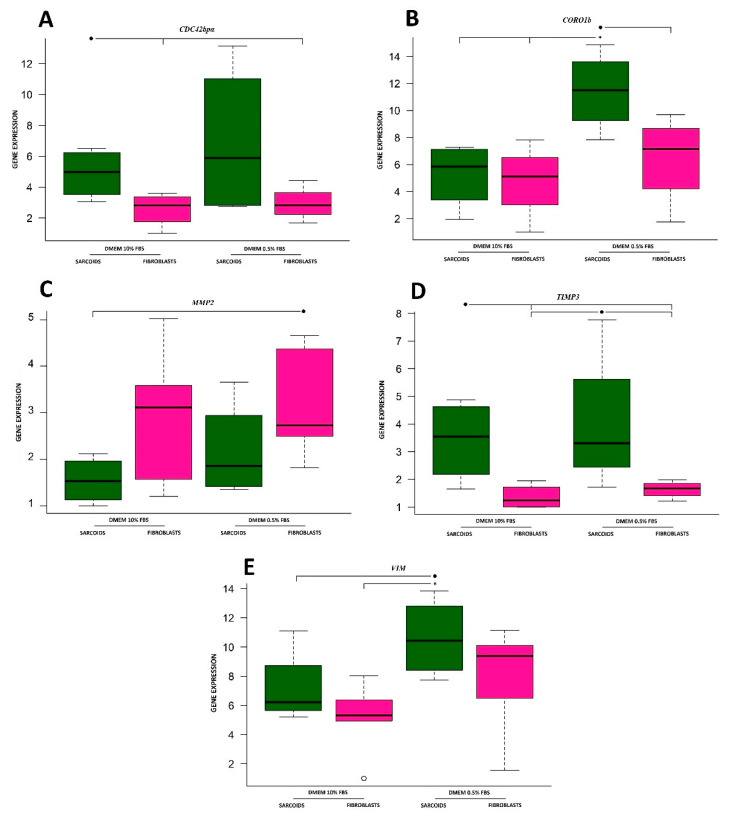
Gene expression patterns of analyzed genes. (**A**) Cell division control 42 binding protein α; (**B**) coronin; (**C**) matrix metalloproteinase 2; (**D**) tissue inhibitor of metalloproteinases 3; (**E**) vimentin. Significant differences (*p* ≤ 0.05) are marked with *, and results showing trends (*p* ≤ 0.1) are marked with ●.

The lowest values of *CDC42bpα* gene expression (Figure 1A) were obtained for fibroblasts, which were two- or three-fold lower than the level in sarcoids, regardless of serum concentration. There were no significant differences in gene expression between fibroblasts and sarcoid cell lines cultured in both medium types. Gene expression in the sarcoid line was two-fold higher than the gene expression in fibroblast cells in the same culture medium (10% FBS). A similar situation was observed for the 0.5% FBS medium.

### 3.2. Coronin

The highest gene expression level of coronin was almost five-fold higher than the other cell cultures in sarcoids cultured in medium containing 0.5% FBS (Figure 1B). The obtained results were significantly different from the results obtained for cell lines cultured in 10% FBS medium, but only for that sample. The rest of the groups had similar coronin expression levels, but higher values were estimated for cells cultured in medium containing less serum.

### 3.3. Matrix Metalloproteinase 2

There were no significant differences in *MMP2* gene expression levels between the analyzed cell lines (Figure 1C).

### 3.4. Tissue Inhibitor of Metalloproteinases 3

The highest values of *TIMP3* gene expression (Figure 1D) were observed in both sarcoid cells. Lower values characterized both fibroblast cell lines in the corresponding serum concentration groups. There were no significant gene expression differences observed.

### 3.5. Vimentin

The obtained results (Figure 1E) showed no significant differences in vimentin gene expression between cells of the same type. The only significant difference was present between the sarcoid cell line cultured with low serum and the fibroblast cell line incubated in culture medium with 10% FBS. The expression values for cell lines incubated in medium with 10% serum showed lower values.

## 4. Discussion

The panel of analyzed genes showed no statistically significant differences between sarcoids and fibroblast cell cultures for three of the analyzed genes: *CDC42bpα*, *MMP2* and *TIMP3*.

The product of the *CDC42bpα* gene exhibits serine-threonine kinase activity and binds to cell division control protein 42 (CDC42), which is a member of the Rho-GTPase protein family that exhibits expression changes in several carcinomas [26]. Its increased activity is connected to an increased metastasis tendency [26]. Stengel and Zheng [27] stated that the CDC42 protein was involved in a variety of cellular processes, such as cell migration, proliferation, cytoskeleton remodeling, cell division and cell cycle. CDC42 is also involved in the translation processes of genes. The vast number of processes regulated by this protein may be the reason for the putative oncogenic character of the protein [27]. Therefore, CDC42bpα may also be considered a carcinogenesis factor. The inhibition of CDC42 leads to actin cytoskeleton reorganization, which is also associated with lowered cell hyperproliferation [27,28]. The high variability of gene expression data obtained from the sarcoid cell line cultured in low serum medium likely resulted in the lack of significant differences between cell lines. Therefore, we cannot approve the overexpression of *CDC42bpα* in sarcoid cells, which could result in the increased inhibition of CDC42 protein functionality, which could confirm our hypothesis of decreased sarcoid cell proliferation and mobility.

The two other genes, *MMP2* and *TIMP3*, are involved in collagen catabolism, which is important in the creation of connections between cells and the basement membrane, in case of collagen IV [29,30]. Catabolism of collagen IV is regulated by the product of the *MMP2* gene, which belongs to the zinc-dependent endopeptidase family gelatinase. The main biological function responsible for the cell metastasis and invasiveness traits is the collagen IV catabolism, which is an important factor in tumor invasion [30]. The results presented in this study do not confirm the results available in the literature [31]. Our results showed no differences in regulation of the analyzed gene in both cell lines. The second gene, *TIMP3*, was upregulated in sarcoid lines compared to fibroblast cells. The product of that gene, tissue inhibitor of metalloproteinases 3, regulates the processes connected to cell adhesion, cell migration and invasion via inhibition of metalloproteinases. Lower *TIMP3* expression is observed in many invasive tumors, and studies performed on tumors influenced by the product of the *TIMP3* gene resulted in reduced cell migration and invasion. Therefore, this protein is considered an oncosuppressor [32,33]. This information may lead to the conclusion that *TIMP3*-up-regulated cells will be characterized by slower migration and invasion ability due to the down regulation of collagen catabolism. Therefore, we conclude that sarcoid cells exhibit less migration and less invasive characteristics than fibroblast cells, which is inconsistent with the available literature [31].

Statistically significant differences in expression were found for *CORO1b* and *VIM*. All of the mentioned genes shared the trait of significant differences between fibroblast cells cultured in 10% FBS medium and sarcoids cultured in 0.5% FBS medium. All results for the low nutrition availability medium (0.5% FBS) were higher than the standard culture medium. This trend indicates that the studied molecular pathways may be overexpressed under low serum conditions, which may be an attempt by cells to move to a new location with abundant nutrients.

Coronin 1b is a protein involved in actin cytoskeleton organization regulation via binding to the actin-related protein 2/3 complex (Arp2/3). Its higher transcript amount has a negative impact on actin nucleation and therefore cell migration [34,35]. Coronin 1b protein enables endothelial cell migration in some cases [35]. Coronin 1b has a positive impact on the creation of aligned actin filament bundles via its interaction with Arp2/3, which enables non-muscle myosin II (NMII) motor proteins to create contractile forces and junctional tension inside cells. NMII is involved in the GTP-RhoA signalling pathway, which means that a lower amount of CORO1b protein in cells results in lower NMII involvement and leads to the inhibition of GTP-RhoA signalling [36]. The higher coronin expression in sarcoid cells (10% FBS) may lead to the conclusion that fibroblasts are more likely to migrate. The results suggest that serum starvation (0.5% FBS) is the factor that inhibits cell migration ability.

Vimentin also encodes a protein related to various traits connected with cell motility. Vimentin is associated with formation of the cytoskeleton, stabilization of cell shape, regulation of signal transduction and cell adhesion, and the absence of this protein results in a lack of strength of cell binding [26,37]. Vimentin is important for the process of directional migration, and its high concentration in specific parts of the cell results in a decrease in the actin filament formation rate [38]. Trials performed in mice confirmed that the lack of vimentin was linked to flawed cell migration [37]. Up-regulated vimentin is strongly related to epidermal-mesenchymal transition (EMT) in epithelial cell cultures, which plays an important role in cancer metastasis [19,37,38]. Notably, vimentin is up-regulated in cell cultures due to an element in the vimentin promoter region, which is responsive to culture media components [38]. Cells in the current study were cultured in the same culture media (DMEM), which enabled comparison. The observed differences in the expression pattern of vimentin may indicate that sarcoid cells, although non-significant for all medium variants, are characterized by a higher expression of the analyzed gene. These results may lead to the conclusion that sarcoid cells are more able to migrate than fibroblasts.

## 5. Conclusions

The results obtained in recent studies may indicate the vast complexity of the genetic background that occurs in sarcoid-affected cells. Obtained data for studied gene patterns indicated tendencies for differential expression between analyzed cell lines. In case of two genes, the results were statistically significant. The results highlight the need of performing further research, which may lead to the creation of sarcoid migration and invasion-related genes panel.

## Figures and Tables

**Table 1:** Sequences of real-time PCR primers and length of the obtained products.

Gene	Primer Sequence	Product Length (bp)
*CDC42bpα*	F:GCTCCATTCAAACGACCACA	176
	R:AAGGATTTGCTGGCCACATC	
*CORO1b*	F:AGATCGCCCGGTTCTACAAA	179
	R:CAGGGAAATGAGGATGGGGT	
*MMP2*	F:TCCCACTTTGATGACGACGA	182
	R:AAGTTGTAGGTGGTGGAGCA	
*TIMP3*	F:AAGATGCCCCATGTGCAGTA	213
	R:TGCAGTTACAACCCAGGTGA	
*VIM*	F:ACAAGTCCAAGTTTGCCGAC	262
	R:CGCGCCATTTCTTTCCTTCAT	
*GAPDH*	F:TCACCAGGGCTGCTTTTAAC	156
	R:GCCTTTCCGTTGATGACAAG	

F—forward primer, R—reverse primer.
